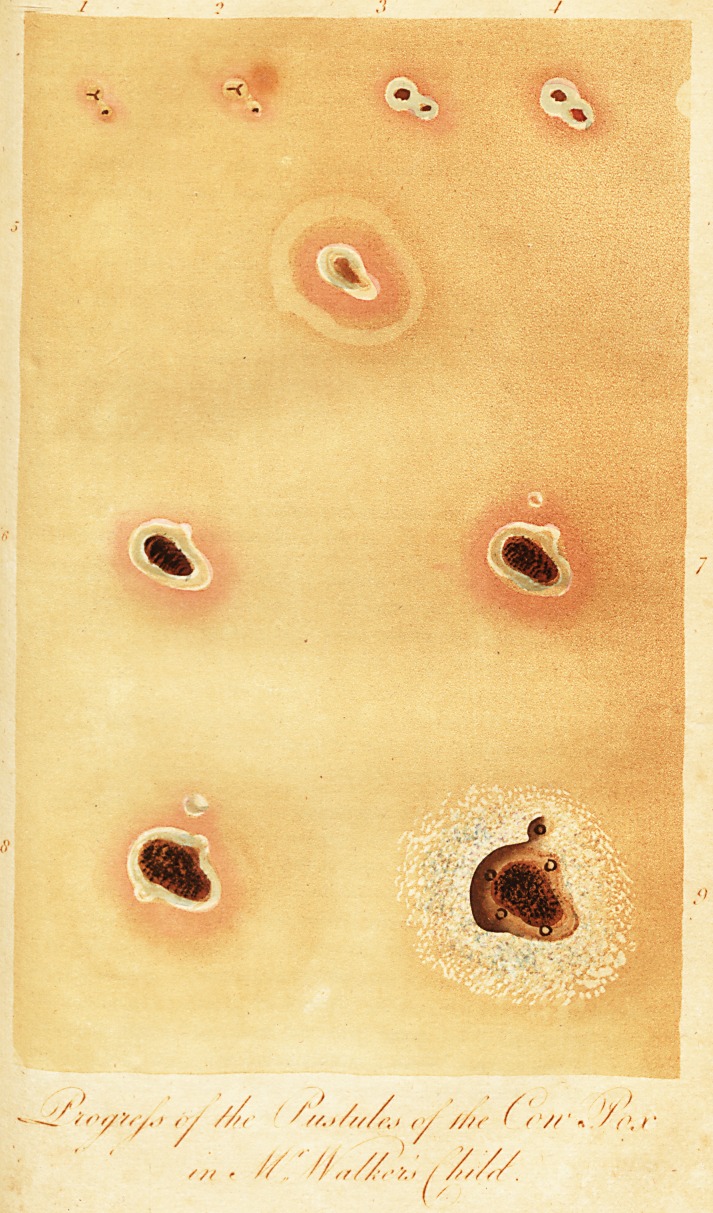# Case of the Cow-Pox, Communicated by Inoculation to His Own Child

**Published:** 1799-04

**Authors:** John Walker

**Affiliations:** Engraver


### *Case of the Cow-pox, communicated by Inoculation to his own Child, by Mr.* JOHN WALKER, *Engraver.*

(With a coloured Plate, representing the progress of the Pustules through their different Stages.)

HAVING a son between ten and eleven months old, and the small-pox being in my opposite, as well as my next neighbour's family, I conceived it so unlikely for my infant to escape the contagion; and having heard of the benign tendency and mildness of the cow-pox, I made it the subject of particular enquiry; and, from all I could learn, there had never been known an instance of its proving fatal. On the other hand, I had experienced the small-pox in my own family, both natural and inoculated, to terminate mortally. On these considerations, and admitting it should not have the desired effect of a preventative against the small-pox, it would still leave my child in the same situation as others; but, if it really were a preservative, as it appears to be in numerous instances, the advantage would be incalculable, and, if encouraged, might in the end even annihilate that dreadful scourge of mankind, the small-pox.

To encourage others, I transmit my observations, made in the progress of the disorder, accompanied with a drawing, exhibiting the various appearances of the pustules.

On Monday, 19th February, 1799, the infection was given by Doctor WOODVILLE, in the presence of Dr. WILLAN, by two incisions, but no material change appeared till the 21st, when the inflammation was increased, and a rising might be felt at each puncture, though not visible, as given in the plate, at No. 1.

On the fourth day, the pustules at the punctures were very distinct, and the inflammation spread, as at No. 2.

On

Progress of the Pustules of the Cow Pox in Mr. Walker's Child.

Publish'd May 1. 1799 by R. Phillips No. 71 St. Pauls Church Yard.

On the ſixth day, the puſtules had run together, with a ſmall brown ſcab depreſſed in the middle, over each inciſion, ſurrounded with a thin ſcale-like edge, too difficult to be repreſented by the engraver; the puſtules were full of a whey-like matter, as at No. 3.

On the ſeventh day, one of the ſcabs was accidentally rubbed off, and the puſtules became a little browner, as at No. 4.

On the ninth day, it was one irregular puſtule; the ſcabs alſo became one, with a faint inflamed circle, detached, as No. 5.

On the 2d of March, the eleventh day, that inflammation had entirely diſappeared, being united; and the ſcab had become much darker and enlarged, with the ſcaly edge before remarked; the circumference of the ſcab leſs regular, and almoſt impoſſible to be repreſented by the engraver. A ſmall puſtule was obſerved, quite white, cloſe to the edge of the large one, and which ſoon ſo united with it, that it gave its edge more irregularity, as No. 6.

On the thirteenth day, another puſtule came out, at a greater diſtance, tranſparent, white, and round, as No. 7.

On the fourteenth day, another puſtule, ſimilar to the former, appeared on the oppoſite ſide of the ſcab. The former had become more whey-like, and the ſkin a little brown; and, while making the drawing that day, the puſtule which I obſerved on the thirteenth day, broke, and a clear tranſparent fluid oozed out, and ſtood on the puſtule, like a drop of dew, in ſize rather bigger than a common pin's head. I alſo found the core or hardneſs, which I had before obſerved under the ſore, was enlarged, and the inflammation much increaſed, both in colour and extent, as No. 8.

On the ſeventeenth day, the puſtule that came out on the thirteenth, and broke on the fourteenth day, was quite dry and brown; the others which had run into the large one, were now more diſtinct than at any time before; as each ſeparate ſcab, of which there were five, although only three puſtules were obſerved, roſe above the others, and the whole had quite a poliſh. The ſcab of the diſtinct puſtule came off, about ten or eleven days after its firſt appearance. The core, on the day after its increaſed ſize was obſerved, was equally perceptible in its decreaſe, and both this and the inflammation gradually abated; the latter becoming rather lived, like a freſh-healed wound when cool. The cuticle all over it ſeemed about to peel off, being cracked, and the white edges giving ſomewhat the appearance of whiting, or flour ſlightly wiped off, as No. 9.

The

The middle, where the firſt ſcabs were formed, was dented in, and of a very dark brown colour, almoſt black, which gradually ſpread over the whole, and the poliſh went off, the ſcab drying, contracted and became dull.

On the 18th of March, the whole ſcab was ſeparating all round the edges, and the child was inoculated with variolous matter for the common ſmall-pox, which, on this day, (March 21) is evidently dying away, like a ſimple ſcratch.

During the whole time, the child never exhibited any particular ſymptoms of indiſpoſition, and had ſo little fever, that it was hardly, if at all, perceptible; he, however, ſeemed by the motion of his arm, to be ſenſible of a ſoreneſs under it; but neither that, nor the eruption affected him ſo much as to render him croſs or peeviſh, although he cut three teeth during the progreſs of the diſorder; he went entirely alone before he was eleven months and a half old.

*Roſamond Street, Clerkenwell,*

*21ſt March,* 1799.

*** *The preceding communication on ſo intereſting a ſubject, we conſider as peculiarly valuable; as Mr.* WALKER *not only ſhews a mind ſufficiently enlightened to give a fair trial to a new method of conquering one of the moſt dreadful ſcourges to mankind, but, from his profeſſional abilities, alſo enables us to exhibit the progreſſive appearances of the cow-pox. We regret, however, that on account of the very late arrival of this paper, the plate alluded to cannot be prepared in time for the preſent Number, but it will certainly appear in the next.*

## Figures and Tables

**Figure f1:**