# Errata

**Published:** 1799-05

**Authors:** 


### ERRATA.

*Page* 119, *line* 9, *for* 'the *inflammation*,' *read*, that *inflammation*.

—— ibid. *l*. 19, *for* '*of the* arm,' *read*, *of the* scab.

